# Epigenetic preconditioning with decitabine sensitizes glioblastoma to temozolomide via induction of *MLH1*

**DOI:** 10.1007/s11060-020-03461-4

**Published:** 2020-03-19

**Authors:** Matthew Gallitto, Rossana Cheng He, Julio F. Inocencio, Huaien Wang, Yizhou Zhang, Gintaras Deikus, Isaac Wasserman, Maya Strahl, Melissa Smith, Robert Sebra, Raymund L. Yong

**Affiliations:** 1 Departments of Neurosurgery and Oncological Sciences, Icahn School of Medicine At Mount Sinai, 1468 Madison Avenue, Box 1136, New York, NY 10029 USA; 2 Department of Neurological Surgery, Albert Einstein College of Medicine, Montefiore Medical Center, New York, NY USA; 3 Department of Genetics and Genomic Sciences, Icahn School of Medicine At Mount Sinai, New York, NY USA

**Keywords:** Decitabine, DNA methylation, Chemosensitization, Glioblastoma, Mismatch repair, Temozolomide

## Abstract

**Introduction:**

To improve the standard treatment paradigm for glioblastoma (GBM), efforts have been made to explore the efficacy of epigenetic agents as chemosensitizers. Recent data suggest possible synergy between decitabine (DAC), a DNA hypomethylating agent, and temozolomide (TMZ) in GBM, but the mechanism remains unclear. The objective of this study was to determine the effects of DAC on TMZ sensitization in a consecutively derived set of primary GBM cultures, with a focus on mismatch repair (MMR) proteins.

**Methods:**

Half maximal inhibitory concentrations (IC_50_) of TMZ were calculated in eleven consecutive patient-derived GBM cell lines before and after preconditioning with DAC. MMR protein expression changes were determined by quantitative immunoblots and qPCR arrays. Single-molecule real-time (SMRT) sequencing of bisulfite (BS)-converted PCR amplicons of the *MLH1* promoter was performed to determine methylation status.

**Results:**

TMZ IC_50_ significantly changed in 6 of 11 GBM lines of varying *MGMT* promoter methylation status in response to DAC preconditioning. Knockdown of MLH1 after preconditioning reversed TMZ sensitization. SMRT-BS sequencing of the *MLH1* promoter region revealed higher levels of baseline methylation at proximal CpGs in desensitized lines compared to sensitized lines.

**Conclusions:**

DAC enhances TMZ cytotoxicity in a subset of GBM cell lines, comprising lines both *MGMT* methylated and unmethylated tumors. This effect may be driven by levels of MLH1 via E2F1 transcription factor binding. Using unbiased long-range next-generation bisulfite-sequencing, we identified a region of the proximal *MLH1* promoter with differential methylation patterns that has potential utility as a clinical biomarker for TMZ sensitization.

**Electronic supplementary material:**

The online version of this article (10.1007/s11060-020-03461-4) contains supplementary material, which is available to authorized users.

## Introduction

Current treatment for glioblastoma (GBM), the most common and aggressive primary malignant brain tumor in adults, consists of maximum surgical resection followed by adjuvant radiotherapy and temozolomide (TMZ) [1]. TMZ is an alkylating agent that induces the formation of methyl adducts, most importantly at the O^6^-guanine position. Methylguanine mispairs with thymine instead of cytosine during replication, which initiates DNA mismatch repair (MMR). A futile cycle of DNA mismatching and attempted repair ensues, resulting in replication fork collapse, DNA strand breaks, and apoptosis [2, 3]. Failure to trigger DNA replication checkpoints, if MMR is deficient, can lead to apoptotic escape and drug resistance [4, 5]. Inactivating mutations and loss of expression of MMR genes in GBM has been correlated with higher tumor proliferation rates and poorer survival outcomes [6–8].

Hypomethylating agents have garnered interest as a means of restoring the expression of genes that might aid anticancer treatment. Decitabine (DAC) is a nucleoside analog that functions by irreversibly binding to DNA methyltransferases (DNMTs), depleting free enzyme, and preventing further DNA methylation during subsequent replication cycles [9]. Due to the high frequency of mutations in DNA methylation enzymes in hematologic malignancies, which cause silencing of tumor suppressors via aberrant hypermethylation, DNMT inhibitors such as DAC have a well-established role in the treatment of patients with myelodysplastic syndrome (MDS) and acute myelogenous leukemia (AML) [10, 11], where they exert their epigenetic effects at relatively low doses (5–20 mg/m^2^/d) [12–16]. In solid malignancies, where driver mutations involving methylation enzymes are uncommon, the ability of DAC to re-express genes that might reduce resistance to cytotoxic agents is of significant interest. Preclinical data showing that the MMR protein mutL homolog 1 (MLH1) can be re-expressed using DAC in ovarian and colon cancer cells to improve sensitivity to platinum agents spurred the development of several clinical trials [17–19]. In advanced melanoma, low-dose DAC was tested in combination with TMZ, yielding an objective response rate of 18% with minimal toxicity [20–22]. A challenge in demonstrating the efficacy of this approach has been the availability of a biomarker to rationally select patients with amenable gene methylation profiles, such as a hypermethylated *MLH1* promoter. In correlative analyses, pre- and post-treatment tissue samples often do not demonstrate the targeted methylation or gene expression change [18, 23].

In GBM, an agent that potentiates TMZ cytotoxicity by increasing MMR activity could be particularly impactful, since TMZ remains the cornerstone of adjuvant therapy. DAC in particular holds promise given its ability to cross the blood–brain barrier to reach cerebrospinal fluid (CSF) concentrations up to 50% of plasma levels [24]. Furthermore, several studies have identified aberrant hypermethylation in the *MLH1* promoter in up to 15% of GBM specimens [25–27], suggesting that a substantial subset of patients might benefit from DAC preconditioning. Published data may underestimate the true rate of hypermethylation of MMR gene promoters due to the use of techniques that limit the number of CpGs profiled in a single assay. There have been three preclinical studies on GBM cell lines demonstrating possible synergy between DAC and TMZ [28–30], but none investigated whether this might be mediated by demethylation of gene promoters causing MMR protein re-expression.

Here, using a set of prospectively derived IDH-wildtype GBM cell lines of mixed *MGMT* methylation status, we sought to evaluate the effects of DAC preconditioning on TMZ sensitivity and MMR protein expression. We leveraged the long-read capabilities of single molecule real-time (SMRT) bisulfite sequencing to profile a 2.5 kb segment of *MLH1* promoter before and after DAC treatment, and identified several loci with potential clinical utility as predictive biomarkers of DAC response.

## Methods

See Online Resource 1 for full details.

### Ex-vivo treatment of GBM spheroid cell lines

For cell lines treated with TMZ after DAC preconditioning, medium containing DAC 100 nM was replenished every 24 h for 5 days. Cells were resuspended in serum-free medium containing TMZ 10 µg/mL (0.05 mM) and 100 nM DAC daily for 2 days. At the completion of concurrent treatment, cells were resuspended in serum-free medium and harvested at 4, 24, 48, and 96 h. A schematic overview of all treatment conditions is provided in Online Resource 2.

### Determination of IC_50_

GBM cell lines were cultured in T25 flasks until 70–80% confluence, and then preconditioned with 100 nM DAC for 7 days; non-treated cells were cultured in parallel. Cells were then digested and resuspended to a final concentration of 2 × 10^5^ cells/mL in Neurobasal Medium (Gibco, #21,103–049). 50 µL of cell suspension was added to 96-well plates (10,000 cells/well) with serial dilutions of TMZ ranging from 0 to 2.5 mM. Plates were incubated at 37 °C for 72 h. Absorbance was recorded at 490 nm. Raw data was normalized to the mean absorbance of the 0 mM TMZ wells. IC_50_ was determined by a nonlinear regression least squares fit for [inhibitor] vs. response (four-variable slope model) using Graphpad Prism 7.0 software.

### Single-molecule real-time (SMRT) sequencing

PCR samples were barcoded and pooled as previously described [31]. SMRT sequencing was performed according to the P5-C3 Pacific Biosciences protocol with a movie collection time of 180 min. Raw sequencing reads in FASTQ format were demultiplexed and trimmed using NGSutils [32], and then aligned to the *MLH1* promoter sequence (hg38) with Bismark and Bowtie2 [33, 34]. The Bismark “coverage2cytosine” script was used to generate an Excel file, from which percent methylation at each CpG site was calculated. Read depth ranged from 500-2500X per sample, depending on multiplexing conditions.

## Results

### DAC sensitizes certain GBM cell lines to TMZ treatment

**Fig. 1:**
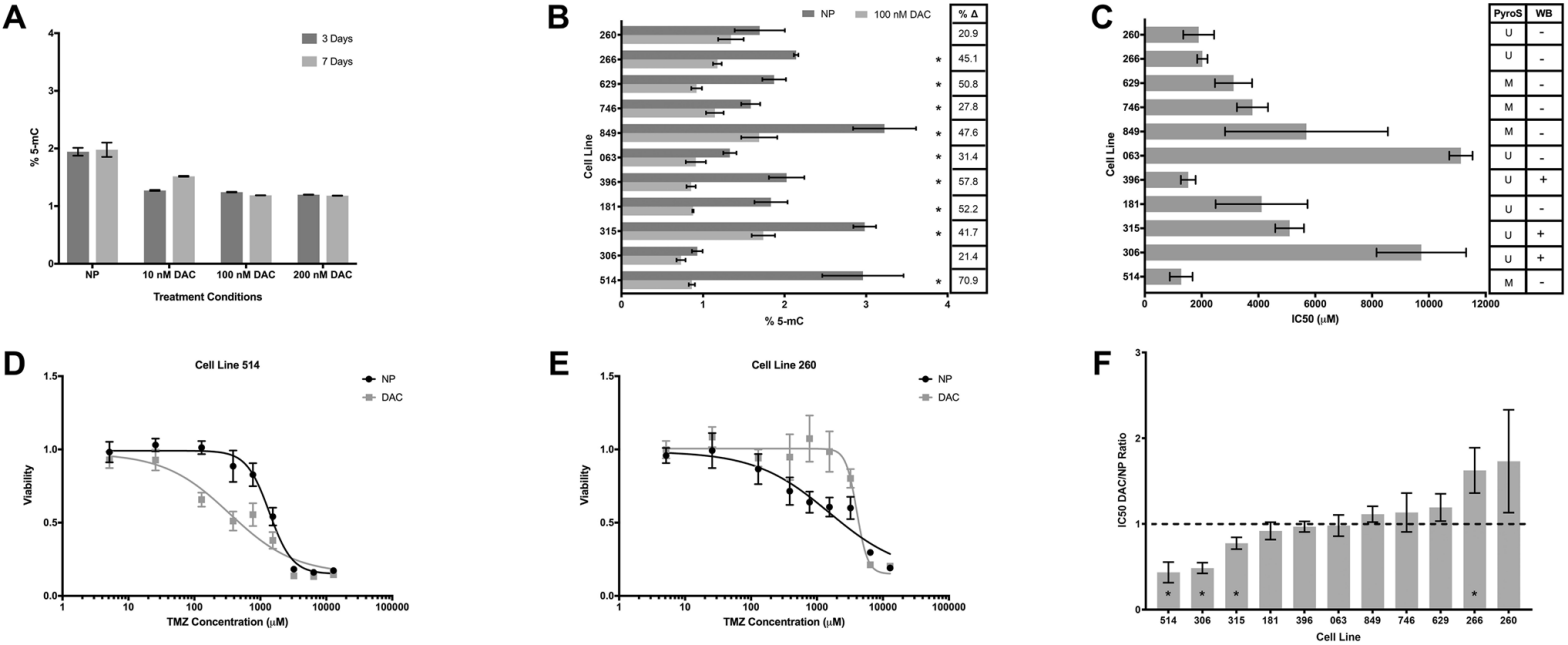
Effect of DAC on genome-wide 5-mC levels and TMZ IC_50_. **a** Mean ± SEM genome-wide 5-mC levels by ELISA for GBM cell line 260 after 10–200 nM DAC treatment for 3 and 7 days (2-way ANOVA, concentration p < 0.0001, length of treatment p = 0.18, interaction p = 0.067). **b** Mean ± SEM genome-wide 5-mC levels by ELISA for eleven GBM cell lines treated with 100 nM DAC for 7 days. Right column indicates relative change from baseline. Asterisks indicate cell lines with significant decrease in 5-mC levels (Student’s *t* test, p < 0.05). **c** Mean TMZ IC_50_ ± SEM in eleven GBM cell lines. Right columns indicate unmethylated (U) or methylated (M) *MGMT* promoter status based on pyrosequencing (PyroS), and MGMT expression on western blot (WB). **d** Dose–response curve for line 514 showing TMZ sensitization with 100 nM/days × 7 days DAC preconditioning. **e** Dose–response curve for line 260 showing TMZ desensitization with 100 nM/days × 7 days DAC preconditioning. **f** Mean DAC/NP IC_50_ ratio ± SEM for eleven GBM cell lines. Dashed line indicates ratio of 1 (neither sensitization nor desensitization). Asterisks indicate significant change in IC_50_ with DAC preconditioning (ratio paired *t* test, p < 0.1)

Using ELISA assays, we determined that 100 nM DAC for 7 days was sufficient to effect genome-wide demethylation without cytotoxicity (Fig. 1a, b). We then calculated the TMZ IC_50_ value for each line based on three independent assays using non-preconditioned (NP) cells of differing passage level (Fig. 1c). To evaluate the effect of DAC on TMZ sensitivity, these IC_50_ values were compared to a matched value obtained from an assay performed simultaneously on DAC-preconditioned cells of identical lineage and passage level, yielding a DAC/NP IC_50_ ratio (Fig. 1d–f). Ratios significantly less than 1, indicating TMZ sensitization, were observed in three cell lines: 514, 306, and 315. Ratios greater than 1, indicating TMZ desensitization after DAC preconditioning, were observed in another three cell lines: 629, 266, and 260. Interestingly, *MGMT* methylated and unmethylated cell lines were distributed proportionately among the two groups, suggesting that determination of *MGMT* methylation status using clinical pyrosequencing protocols would not provide sufficient information to predict the effect of DAC preconditioning on TMZ sensitivity.

### DAC-sensitized GBM cell lines exhibit increased MLH1 expression

**Fig. 2:**
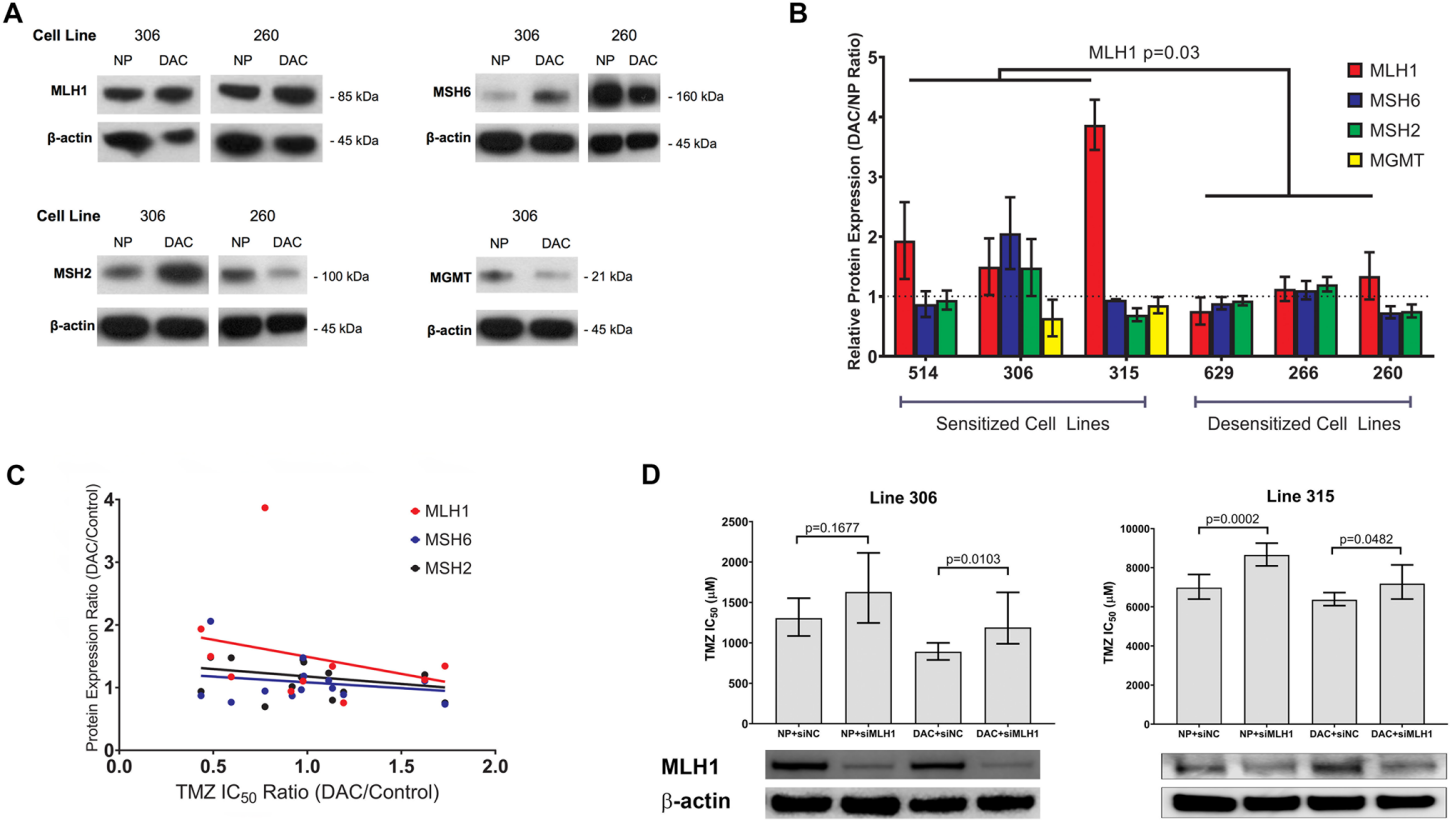
Changes in MMR and MGMT protein expression in GBM cell lines after DAC treatment. **a** Immunoblots of MLH1, MSH2, MSH6 and MGMT in sensitized line 306 and desensitized line 260. **b** Immunoblot band intensities were quantified and normalized against β-actin. Expression levels in DAC-preconditioned and NP cells from the same line were then compared. The mean DAC/NP ratio ± SEM for each sensitized and desensitized line is shown. The dashed line indicates a DAC/NP ratio of 1 (protein level unchanged by DAC). Average MLH1 DAC/NP ratio is 2.31 compared to 1.12 in sensitized versus desensitized cell lines (two group *t* test, p = 0.03). **c** DAC/NP MLH1, MSH2, and MSH6 protein expression ratios wer plotted against the DAC/NP IC_50_ ratio for all cell lines. For MLH1, r =  − 0.62 and p = 0.077, Spearmann’s rank test. **d** Two cell lines, 306 and 315, exhibiting TMZ sensitization, were unpreconditioned (NP) or preconditioned with DAC (DAC) for 7 days. During the last 2 days of preconditioning, cells were transfected with 10 nM scrambled (siNC) or MLH1-specific (siMLH1) siRNA. TMZ IC50 was then determined using MTS assays and compared using extra sum-of-squares F tests

**Fig. 3:**
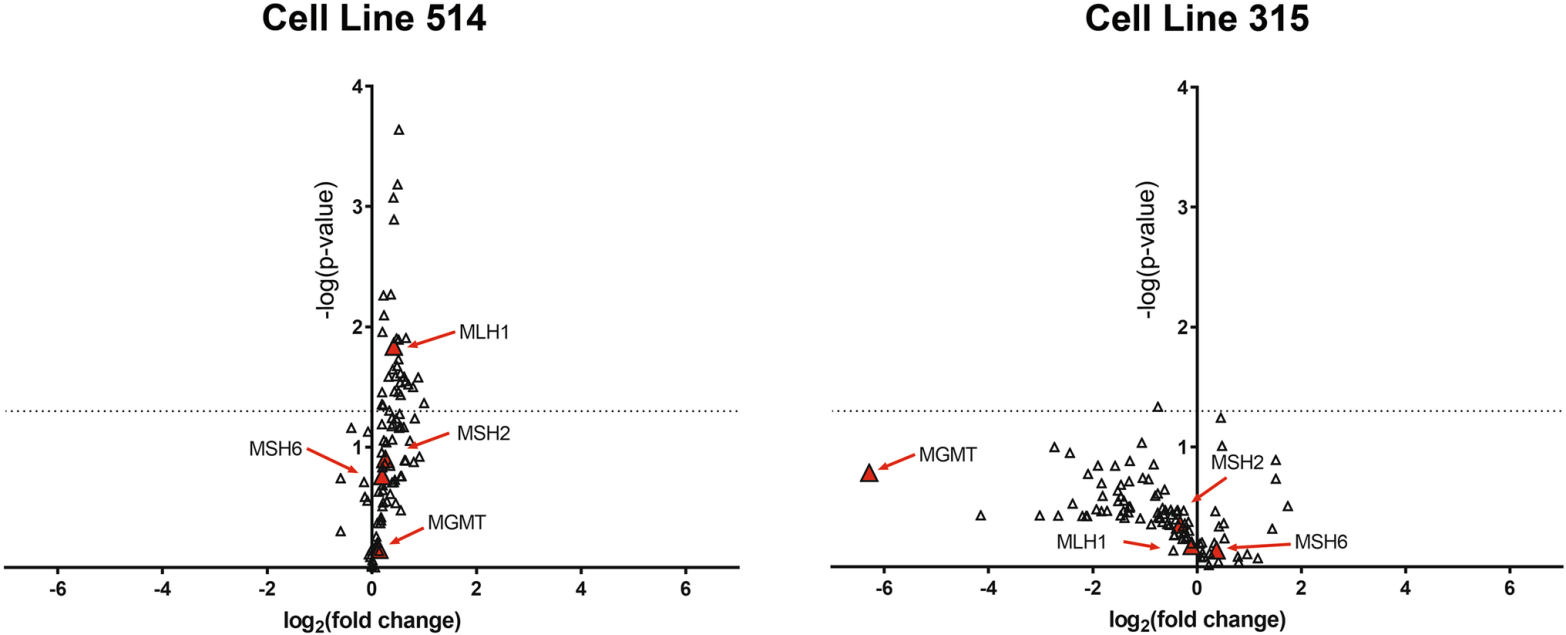
Volcano plots indicating significance and fold-change of mRNA expression level of 84 DNA damage repair genes in two TMZ sensitized cell lines (315 and 514) after DAC preconditioning. Dashed line indicates p = 0.05

We next investigated how DAC altered MMR protein expression in sensitized versus desensitized lines. Levels of MLH1, MSH6, and MSH2 were measured in each of the six cell lines after treatment with 100 nM of DAC for 7 days and compared to its untreated control using quantitative immunoblots (Fig. 2a, see Online Resource 1 for methods). Using a mutation hotspot panel, no clonal MMR gene variants were detected in any line (see Online Resource 1). At baseline, MLH1 level was significantly negatively correlated with TMZ IC_50_ (r =  − 0.8214, p = 0.0341, Spearman’s rank test). In the three TMZ-sensitized cell lines MLH1 levels increased 1.5- to fourfold, while no significant increases or decreases were detected in the three TMZ-desensitized cell lines (Fig. 2b). Correlating the IC_50_ DAC/NP ratios with MLH1 DAC/NP expression ratios for ten cell lines on which full data were available, there was a negative correlation that trended towards significance (r =  − 0.62, p = 0.077, Spearman’s rank test) (Fig. 2c). The effect of DAC on the expression of the other functionally important MMR proteins MSH6 and MSH2 was more variable. At baseline, levels for both proteins had a negative correlation with TMZ IC_50_, with MSH2 trending toward significance (r =  − 0.5636, p = 0.0761, Spearman’s rank test). However, no significant trends were identified when correlating the IC_50_ DAC/NP ratios with MSH6 and MSH2 DAC/NP expression ratios in thirteen cell lines. Using cell line(s) 315 and 306, siRNA knockdown of MLH1 abrogated the TMZ-sensitizing effect of DAC, pointing to a causal relationship between MLH1 and TMZ chemosensitivity (Fig. 2d). Orthogonal validation using qPCR arrays confirmed an increase in *MLH1* expression by 1.3-fold in line 514 and no changes in *MSH2* or *MSH6* (Fig. 3). We did not identify any other DNA repair genes that were significantly up- or downregulated by DAC in both cell lines (Online Resource 3).

### Relationship between TMZ sensitization and MGMT expression

We next asked whether MGMT expression changes correlated with the degree of TMZ sensitization observed. Among the three TMZ sensitized lines, the *MGMT* unmethylated lines 306 and 315 concordantly exhibited detectable baseline MGMT expression, which decreased significantly with DAC treatment (Fig. 2b). This was corroborated by qPCR array data showing a dramatic log_2_ fold-change of − 6.29 for MGMT in line 315 (Fig. 3). The third sensitized line 514 was derived from methylated tumor tissue and concordantly exhibited no MGMT expression at baseline. After treatment with DAC, MGMT expression remained undetectable. Among the three TMZ desensitized lines, none were found to express MGMT at baseline or after DAC treatment despite only one line (629) being predicted to lack MGMT expression based on pyrosequencing. Together, these results indicate that in both TMZ sensitized and desensitized cell lines, MGMT expression did not increase due to DAC treatment, as might be expected, even if the MGMT promoter was hypermethylated.

### TMZ sensitization by DAC is mediated through an intact MMR pathway

**Fig. 4:**
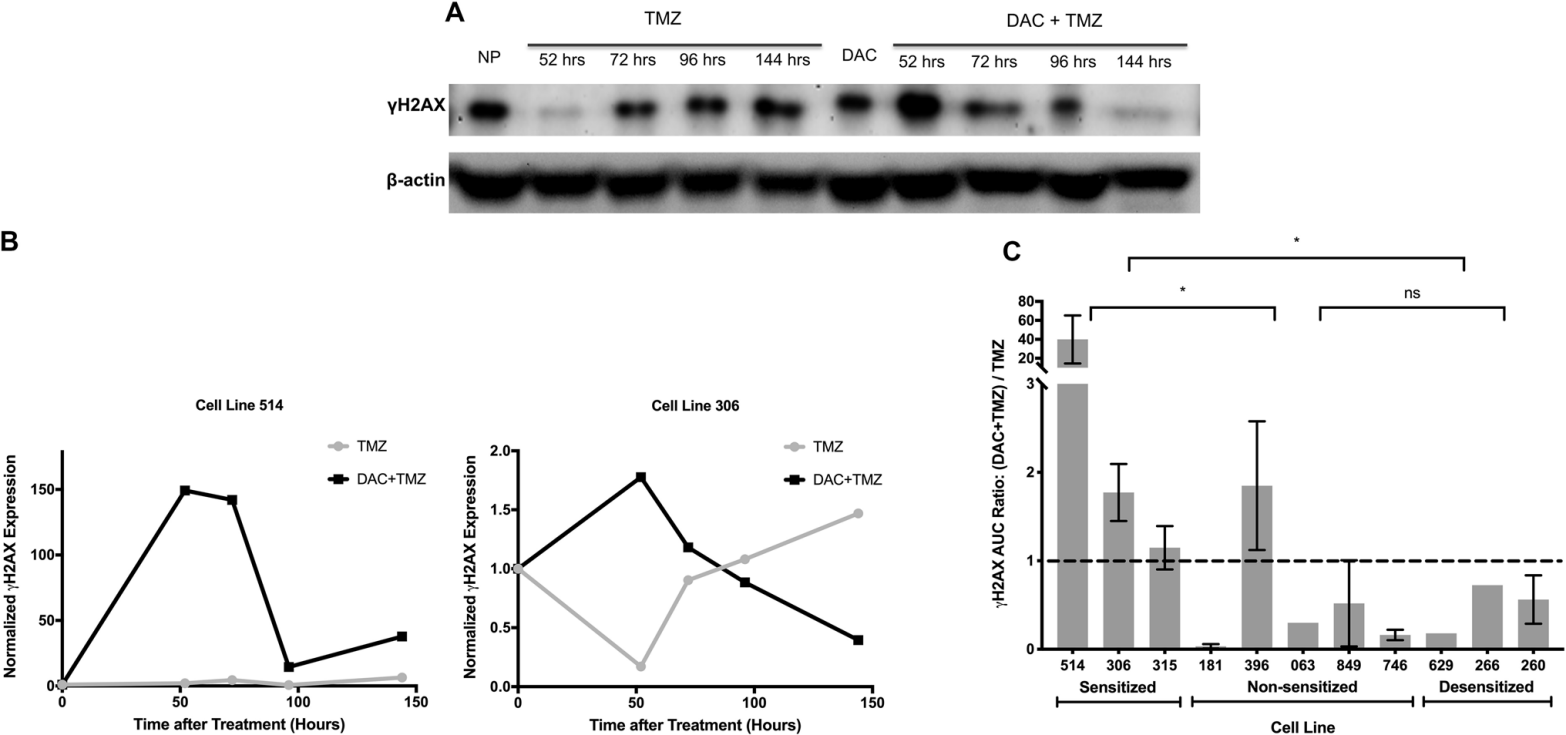
TMZ-induced DNA DSBs in DAC-preconditioned and non-preconditioned GBM cells. **a** Cell line 306 was treated with TMZ alone or with DAC-preconditioning, and immunoblots for γH2AX were performed on whole-cell lysates at the time intervals shown after starting TMZ treatment. **b** Quantification of (**a**) using geometric normalization to β-actin and the γH2AX level before TMZ treatment is shown for sensitized lines 315 and 306. **c** The area under the curve (AUC) of γH2AX time course plots in (**b**) was calculated. The mean DAC/NP AUC ratio ± SEM is shown for eleven GBM cell lines. Asterisks indicate significant difference in DAC/NP AUC ratio between the cell line groups (Mann Whitney test, p < 0.05)

Having established that an increase in MLH1 expression was associated with DAC sensitization to TMZ, we sought to assess the functionality of the MMR pathway by measuring DNA double strand break (DSB) formation. Histone H2AX phosphorylation (γH2AX) accumulates at DSBs within minutes and is a surrogate marker for the effects of alkylating agents and radiotherapy [35]. We assessed levels of γH2AX in DAC-preconditioned and NP controls at time points up to 144 h after TMZ exposure (Fig. 4a, b), and compared cumulative γH2AX using area-under-the curve ratios (Fig. 4c). After preconditioning, two of the three TMZ sensitized lines, 514 and 306, showed significantly elevated cumulative γH2AX at 144 h. The third sensitized line, 315, showed no significant difference in γH2AX expression despite having the highest DAC-induced upregulation of MLH1, which may have been due to a concomitant decrease in MSH2. After DAC preconditioning, cumulative γH2AX expression after TMZ was significantly decreased in all desensitized lines. This coincided with reductions in MLH1, MSH2, and MSH6. Our results suggest that, by altering MMR protein levels, particularly MLH1, DAC can potentiate or abrogate DSB formation in GBM cell lines, which is the expected mechanism of TMZ cytotoxicity if MMR is functionally intact.

### Low CpG methylation levels at the proximal MLH1 promoter region correlate with DAC sensitization

**Fig. 5:**
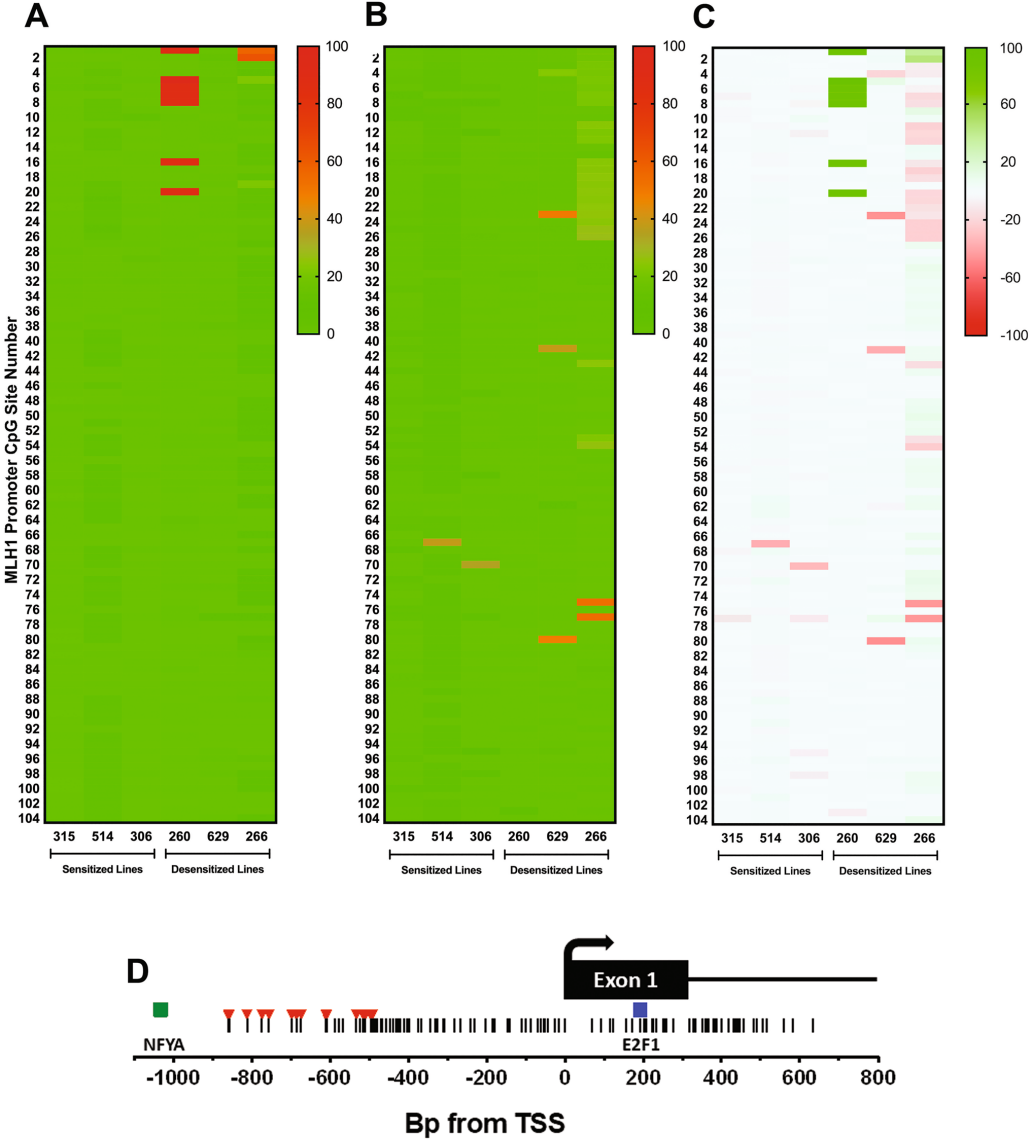
Effect of DAC treatment on *MLH1* promoter methylation in three sensitized and three desensitized GBM cell lines. **a** Long-read SMRT-seq of bisulfite-converted amplicons of a 2.5 kb segment of the *MLH1* promoter was used to quantify the percentage of methylated reads at 104 consecutive CpG sites. Compared to TMZ sensitized lines, desensitized lines showed higher levels of baseline methylation in the region containing CpGs 1–20. **b** Percentage of methylated reads after treatment with DAC 100 nM/days × 7 days. **c** Absolute change in percentage of methylated reads after DAC treatment, with demethylation depicted in green and hypermethylation depicted in red. **d** Schematic of the genomic region containing the CpG island at the *MLH1* promoter, showing the locations of CpG sites (vertical lines), hypermethylated cytosines in desensitized lines (red), and predicted nearby transcription factor binding sites (squares) in relation to the *MLH1* transcription start site (TSS). Genomic coordinate of the TSS is chr3:36,993,350 (hg38)

We next investigated whether the DAC-induced changes in MLH1 levels in TMZ-sensitized and desensitized GBM cell lines could be explained by CpG methylation changes at the *MLH1* promoter. We used long-read real-time sequencing of bisulfite-converted PCR amplicons (SMRT-BS) to quantify methylation levels at 104 consecutive CpGs within a 2.5 kb segment of the *MLH1* promoter region in the three sensitized and three desensitized cell lines. In NP controls, desensitized lines displayed significantly higher levels of methylation across multiple CpGs in the upstream region of the promoter (− 860 to − 492 bp from the transcription start site) compared to sensitized lines (Fig. 5a). In particular, CpG 5 (hg38,chr3:36,992,594) was hypermethylated above 20% in NP controls for all three desensitized lines, but exhibited less than 2% methylation in all sensitized lines. In comparing cells treated with DAC to their NP controls (Fig. 5b, c), there did not appear to be a specific region within the promoter common to all sensitized lines that exhibited significant CpG demethylation. Together, these results suggest that hypomethylation of the proximal promoter region may be necessary for *MLH1* upregulation to occur after DAC preconditioning, and that DAC does not exert its effects through promoter demethylation per se. To further explore the possibility that DAC increases *MLH1* expression indirectly by upregulating one or more transcription factors, using oPOSSUM [36] we performed a binding motif analysis on DAC co-upregulated genes detected in the sensitized lines 315 and 514 via qPCR array. This revealed E2F1 binding sites to be significantly over-represented (− log[p] = 9.528, Fisher’s exact test) in the promoter regions of DAC co-upregulated genes, compared to a control set of 24,752 genes (Online Resource 4). Indeed, a canonical E2F1 binding motif (5′-TTTGGCGC) is present within the first exon of *MLH1* (Fig. 5d).

## Discussion

In this study, we demonstrated that DAC 100 nM for 7 days induces genome-wide DNA hypomethylation in a set of prospectively collected, IDH-wildtype GBM cell lines grown in serum-free conditions. Existing pharmacokinetic data indicate that CSF concentrations in the 100 nM range would be potentially achievable with intravenous DAC in the well-tolerated low dose range [24, 37]. We compared changes in the level of the MMR proteins MLH1, MSH2, and MSH6 before and after DAC treatment, and found that levels of MLH1 most strongly correlated with baseline resistance, and degree of sensitization to, TMZ. Furthermore, MLH1 knockdown was able to reverse the effects of DAC. Previous studies have established the important role MMR deficiency plays in recurrent GBM. The MutSα complex, composed of MSH2 and MSH6 heterodimers, binds to methylguanine-thymine mismatches, and then recruits the MutLα complex, composed of MLH1 and PMS2 heterodimers, to initiate base excision. Although complete deficiency of MMR, which confers the microsatellite instability phenotype, is rare, inactivating mutations acquired during TMZ and reductions in MMR protein expression are common [38, 39]. The relative importance of deficiencies in the MutSα versus the MutLα complex in GBM is less clear. In an analysis of 43 matched pairs of pre- and post-treatment GBM samples, Felsberg et al., saw significant reductions in expression of MSH2, MSH6, and PMS2, but not MLH1 [39]. In a mouse xenograft model of human GBM cell lines, McFaline-Figueroa et al. found that MSH2 knockdown conferred TMZ resistance more potently than MSH6 knockdown [40]. In vitro experiments using U251 cells demonstrated that reductions in MLH1 expression drive destabilization of its binding partner PMS2, and may be more correlated with TMZ resistance than either MSH2 or MSH6 [41, 42]. Our results are overall consistent with the preclinical studies pointing to the relative importance of MLH1.

Interestingly, we observed DAC-induced upregulation of MLH1and TMZ sensitization in both *MGMT* methylated and unmethylated tumors. Of the two unmethylated tumors, one (315) was derived from an aggressive secondary gliosarcoma, and the other (306) from a GBM with a high TMZ IC_50_ of 9.5 mM that decreased by half to 4.7 mM with DAC preconditioning. Identification of *MGMT* promoter methylation status at the time of surgery is routinely used to guide adjuvant treatment on the premise that MGMT expression predicts TMZ responsiveness and improved survival [43]. The prognosis for elderly patients with *MGMT* unmethylated tumors is particularly poor [44–46]. Because *MGMT* is unmethylated in 60% of IDH-wildtype GBM, a strategy to chemosensitize GBM using DAC, so that TMZ has wider utility in this subtype, could have a large impact in the poorest prognosis patients.

One theoretical concern is that DAC might act at a hypermethylated *MGMT* promoter to increase expression of MGMT and thus resistance to TMZ. Although significant TMZ desensitization was seen in three GBM cell lines in our study, including one *MGMT* methylated line, no associated increase in MGMT levels was observed. Rather, DAC tended to decrease MGMT levels in TMZ sensitized cell lines, which suggests that DAC alters MGMT expression through other mechanisms. Moen et al. examined the role of gene body methylation levels in MGMT regulation and found that in the presence of an unmethylated promoter, DAC could decrease MGMT expression by demethylating a region of the gene body [28]. They further suggested that gene body methylation status should be considered together with promoter methylation status to improve the prediction of TMZ response. Our findings of the discordant lack of MGMT expression by western blot in 4 of 7 GBMs determined by pyrosequencing to be unmethylated, and the reduction of MGMT levels by DAC in unmethylated lines, lend support to these conclusions.

Previous studies examining the promoter methylation status of MMR genes in GBM cell lines found low rates of aberrant hypermethylation and an unclear relationship between this and treatment response. *MLH1* promoter hypermethylation rates ranging from 2 to 15% have been reported using short-read pyrosequencing [39] and older qualitative assays [25–27]. Rodriguez-Hernandez et al. found that hypermethylation of the proximal *MLH1* promoter region was predictive of loss of protein expression but not for treatment response [26], while Fukushima et al. found that hypermethylation of the distal promoter strongly predicted response to nimustine [25]. To clarify these findings, we turned to a long-read bisulfite sequencing method capable of surveying the entire *MLH1* promoter without the need for PCR subcloning, and report the largest amplicon successfully analyzed using this method to date. Our results corroborate the finding that the proximal promoter region may be critical for MLH1 expression. With DAC, hypermethylation in desensitized lines decreased inconsistently, while the proximal promoter remained uniformly hypomethylated in sensitized lines, suggesting that hypomethylation of this region is necessary but not sufficient for MLH1 expression. This is contrary to previous findings in ovarian and colon cancer xenografts suggesting that upregulation of *MLH1* with DAC is mediated directly by its action at hypermethylated CpGs in the promoter [17]. We speculate that DAC may act indirectly on *MLH1* in GBM by increasing the expression of proapoptotic E2F1 [47], the action of which is blocked by a hypermethylated proximal promoter. Resistance to DAC-mediated demethylation at the proximal promoter could be due to variability among different cell lines in the rate of incorporation of DAC into DNA, which is dependent on nucleoside receptor uptake, pyrimidine metabolism, and the rate of cell cycling [48]. Despite these lingering questions, our findings nevertheless point to the existence of baseline *MLH1*methylation differences between DAC responsive and non-responsive tumors that could see utility as a biomarker for patient selection in future clinical trials.

## Conclusion

DAC preconditioning enhances TMZ cytotoxicity in a subset of GBM cell lines independent of *MGMT* promoter methylation status. This effect appears to be driven by increased expression of MLH1, leading to potentiation of MMR activity and increased DSB formation. We identified an unmethylated region of the *MLH1* promoter, common to sensitized cell lines in their treatment-naive state, which merits further investigation as a clinical biomarker for DAC patient selection. Additional studies will be needed to confirm whether DAC increases functional MLH1 levels through E2F1 transcription factor upregulation.

## Electronic supplementary material

Below is the link to the electronic supplementary material.

Supplementary file 1 (DOCX 43 kb)

Supplementary file 2 (TIF 846 kb)

Supplementary file 3 (XLSX 24 kb)

Supplementary file 4 (XLSX 24 kb)
