# Supplementary material for: Epigenetic preconditioning with decitabine sensitizes glioblastoma to temozolomide via induction of MLH1
Source: J Neurooncol. 2020 Mar 19;147(3):557–66. doi: 10.1007/s11060-020-03461-4 (PMC7256087; doi:10.1007/s11060-020-03461-4)
Supplement: Supplementary file 1 — (DOCX 43 kb) [file 11060_2020_3461_MOESM1_ESM.docx]

**Supplementary Methods**

***Establishment and selection of GBM spheroid cell lines***

All GBM cell lines used in this study were derived from patients enrolled in our institutional IRB-approved cancer biorepository tissue procurement protocol. Resected tumor tissue was processed for clinical diagnosis according to clinical standard operating procedures. A diagnosis of GBM was established according to the criteria of the WHO Classification of Tumors of the Central Nervous System. In addition, our standard diagnostic panel includes EGFR and p53 immunoreactivity, EGFR amplification by in situ hybridization, and MGMT promoter methylation status by pyrosequencing. Consecutive IDH-wildtype GBMs procured by the senior author over a one-year period, which gave rise to robust cultures under serum-free conditions, were selected for further experimentation.To establish cultures, freshly resected tumor tissue was washed and digested as previously described.[1] After centrifugation, cells were resuspended in 6 mL NeuroCult NS-A Proliferation Medium (Stemcell) supplemented with recombinant human EGF (20 ng/mL), and 0.0002% heparin, and then cultured in T25 tissue culture flasks. The medium was changed at 48 hours and subsequently every 3-4 days. All experiments were performed at passage 20 or lower.

***Global 5-methylcytosine quantification***

Genomic DNA extraction was performed using the Purelink Genomic DNA Mini kit (Thermo Fisher Scientific) according to the manufacturer’s protocol. 100 ng genomic DNA was used to quantify global DNA methylation levels using the 5-mC DNA enzyme-linked immunosorbent assay (ELISA) kit (Zymo Research, #D5325). After coating 96-well plates with denatured, single-stranded DNA samples in triplicates, anti-5-methylcytosine monoclonal and horseradish peroxidase-conjugated secondary antibodies were added sequentially and absorbance was measured at 425 nm using a BioTek Synergy H1 Hybrid Multi-Mode Reader. A standard curve was generated from control samples of known 5-mC percentages. Absorbance for controls were plotted as a function of absorbance (y-axis) vs. %5-mC (x-axis) and using the following equation derived from the logarithmic second-order regression, % 5-mC for each DNA sample was determined based on absorbance.

$$\%5mC = e^{\left\{ \frac{Absorbance - y-intercept)}{Slope} \right\}}$$

***Semiquantitative immunoblot***

Total protein was extracted from cell pellets using Cell Lysis Buffer (Cell Signaling, #9803). The protein concentration was quantified using Qubit Protein Assay Kit (Thermo Fisher, Q33211). Equal amounts of total protein for each sample were separated on a NuPAGE 4–12% Bis-Tris gel (Invitrogen) and transferred to a polyvinylidene difluoride membrane (Invitrogen). Membranes were blocked in 5% BD Difco Skim Milk at room temperature for 1 hour before incubation with primary antibodies at 4°C overnight. The following primary antibodies were used: MGMT (Cell Signaling, #2739, 1:1000 dilution), MLH1 (Cell Signaling, #4256, 1:1000 dilution), MSH2 (Cell Signaling, #2850, 1:1000 dilution), MSH6 (Cell Signaling, #12988, 1:1000 dilution), γH2AX (Cell Signaling, #9718, 1:1000 dilution), and β-actin (Cell Signaling, #3700, 1:2000 dilution). Horseradish peroxidase-conjugated anti-mouse or anti-rabbit secondary antibody (Santa Cruz Biotechnology, sc-2004 and sc2005, 1:5000 dilution) was then applied to the immunoblots for 1 hour at room temperature. The blots were visualized using the enhanced chemiluminescence system (Thermo Scientific). The photographic film of each blot was digitized then processed in ImageJ software for band quantification using the “gels submenu” tool (https://imagej.nih.gov/ij/docs/menus/analyze.html#gels). Briefly, a lane profile plot was generated for each band. Then, the straight line detection tool was used to draw base lines enclosing each peak of interest. Finally, the wand tool was used to measure peak areas. Relative band density was calculated by dividing each band’s peak area (intensity) by its corresponding β-actin control band:

$$MLH1 = \frac{MLH1 intensity}{\beta actin intensity}$$

Relative density ratios (DAC/NP) for MLH1, MSH2, and MSH6 were correlated with IC_50_ DAC/NP ratios using Spearman’s rank test for nonparametric data. Multiple experimental values for each cell line were averaged together for the analysis. For comparing γH2AX over time, relative band intensities at four different time points (52, 72, 96, and 144 hours post-TMZ exposure) were plotted, and resulting area under the curve (AUC) values were calculated.

***Knockdown of MLH1 in GBM spheroid cell lines***

Cell lines were preconditioned with 100 nM DAC for 5 days until transfection was performed; non-treated cells were cultured in parallel. On Day 6, cells were transfected with three different sets of MLH1 siRNA (Ambion, s224048, s224047, s533349, each 10 nM) or scrambled negative control (Invitrogen #462001, 10 nM) using Lipofectamine 2000 transfection reagent (Invitrogen, # 13778150). On Day 8, cells were suspended and plated into to 96-well plates. Serial dilutions of TMZ ranging from 0-2.5 mM were added to the cells. IC50 was determined after incubation at 37 °C for 72 hours.

***Targeted IonTorrent DNA sequencing***

Genomic DNA extraction was conducted using Purelink Genomic DNA Mini kits (Thermo Fisher Scientific) to yield ample material for NGS. The Ion AmpliSeq Oncomine Comprehensive research panel version 2.0 (OCP v2.0, Thermo, #4475346) was used to characterize relevant genes at high coverage (~2000X depth) for variant characterization purposes using a pool of multiplexed primers that amplify the targeted loci of frequent mutations in known oncogenes and tumor suppressor genes. After amplification, the libraries were prepared with the Ion AmpliSeq Library Kit 2.0 (Thermo, #4475345) using Ion Xpress Barcode Adapters 1-96 Kit (Thermo, #4474517). A 1.5X bead purification was performed with Agencourt AMPure XP Reagent (Beckman Coulter, #A63880) following the instructions in the Ion Ampliseq Library Prep protocol to clean up the sample and remove adapter dimers. All samples were quantified with the Ion Library TaqMan Quantitation Kit (Thermo, #4468802) using a 1:100 dilution of each library in a 10 μL reaction volume, with 2 technical replicates per sample. Following quantification, all samples were individually normalized to 100 pMol and 2 μl of each normalized library were combined to form a pool of 96 uniquely barcoded samples at a final concentration of 100 pMol. 25 μl of this pool was used for priming the sequencing chip. Ion Torrent chip priming and sequencing were carried out using the Ion Torrent S5XL system and the Ion Chef instrument with reagents from the Ion 540 Kit-Chef (Thermo, #A27759). The Chef was used to bind each library DNA fragment to Ion Sphere Particles (ISPs) and clonally amplify each fragment by emulsion PCR. Amplified DNA fragments were then bound to streptavidin-coated beads and template negative ISPs were washed away. Template-bound ISPs were then prepared for sequencing by loading onto one Ion Torrent S5 540 chip for sequencing on the Ion S5XL sequencing system. The primed 540 chip was sequenced on the Ion Torrent S5XL System with library read length set at 200 bp and 520 flows per chip, with all other instrument settings set to the manufacturer’s default for the Ion 540 Kit. Analyses of sequencing raw data were performed with Ion Torrent Suite (version 5.0.2) using the “coverageanalysis” and “variantcaller” plugins (with somatic/low stringency settings for the “variantcaller”), with all other settings for the run report set to the manufacturer’s default.

***Quantitative PCR array***

Total RNA were isolated from non-preconditioned and preconditioned cells using RNeasy Mini Kit (Qiagen, #74104). 600 ng of total RNA was reverse transcribed into cDNA using RT^2^ First Strand Kit (Qiagen, #320401). The cDNA was then added to the RT^2^ SYBR Green qPCR Mastermix (Qiagen, #330522) and loaded onto the RT^2^ Profiler Array Human DNA Repair (Qiagen, #330231). Real-time PCR was performed using the The StepOnePlus Real-Time PCR System (Thermo Fisher, #4376600) with RT2 SYBR Green qPCR Mastermix (Qiagen, #330500) according to the manufacturer’s protocol. The relative amount of mRNA was determined using the 2-ΔΔCT method, normalized with respect to endogenous GAPDH mRNA expression. Fold change greater than 1.5 with p<0.05 (Student’s *t-*test) was considered significant.

***Bisulfite conversion and PCR***

500 ng genomic DNA was used for bisulfite conversion using the Methylamp DNA Modification Kit (Epigentek, #P-1001). 100 ng bisulfite-converted DNA was amplified using TaKaRa EpiTaq Hot-Start DNA Polymerase (Clontech, #R110). We used a nested PCR approach to maximize yield without compromising specificity. For controls, non-bisulfite converted genomic DNA was amplified using TaKaRa Taq Hot Start DNA Polymerase (Clontech, #R007). The reaction conditions were as follows:

Reaction #1:

| EpiTaq HS (5 U/μL) | 0.125 μL |
| --- | --- |
| dNTP Mixture (2.5 mM each) | 3 μL |
| 10X EpiTaq PCR Buffer (Mg2+ free) | 2.5 μL |
| 25 mM MgCl2 | 3 μL |
| Sense Primer (10 μM) | 1 μL |
| Antisense Primer (10 μM) | 1 μL |
| Bisulfite-converted DNA template | 5 μL (appx. 75 ng) |
| Sterile distilled water | To 25 μL |

Reaction #1 PCR Conditions:

94°C: 2 min

94°C: 20 sec

35 cycles

48°C: 45 sec

65°C: 2 min 35 sec

65°C: 8 min

4°C: ∞

PCR product was purified using QIAquick PCR Purification Kit and eluted with 30 μL sterile distilled water. This product was then used as template for reaction #2.

Reaction #2:

| EpiTaq HS (5 U/μL) | 0.125 μL |
| --- | --- |
| dNTP Mixture (2.5 mM each) | 3 μL |
| 10X EpiTaq PCR Buffer (Mg2+ free) | 2.5 μL |
| 25 mM MgCl2 | 3 μL |
| Sense Primer (10 μM) | 1 μL |
| Antisense Primer (10 μM) | 1 μL |
| Reaction #1 PCR purified product | 3 μL |
| Sterile distilled water | To 25 μL |

Reaction #2 PCR conditions:

94°C: 2 min

94°C: 20 sec

35 cycles

48°C: 45 sec

65°C: 2 min 20 sec

65°C: 8 min

4°C: ∞

Final PCR product was purified using QIAquick PCR Purification Kit and eluted with 30 μL sterile distilled water.

For control purposes, non-bisulfite converted genomic DNA was amplified using TaKaRa Taq Hot Start DNA Polymerase (Clontech, #R007) using the following reaction conditions:

| TaKaRa Taq HS (5 U/μL) | 0.125 μL |
| --- | --- |
| 10X PCR Buffer | 2.5 μL |
| dNTP Mixture (2.5 mM each) | 2 μL |
| Sense Primer (10 μM) | 1 μL |
| Antisense Primer (10 μM) | 1 μL |
| Non-converted genomic DNA | 100 ng |
| Sterile distilled water | To 25 μL |

Non-converted PCR conditions:

94°C: 2 min

94°C: 20 sec

35 cycles

56°C: 45 sec

65°C: 2 min 20 sec

65°C: 8 min

4°C: ∞

Final PCR product was purified using QIAquick PCR Purification Kit and eluted with 30 μL sterile distilled water.

***MLH1 primers***

| **Primer** | **Sequence (5’ 🡪 3’)** |
| --- | --- |
| Bisulfite PCR reaction #1 Forward Primer | TTAAGTGAAGAAATTTTGAATAA |
| Bisulfite PCR reaction #1 Reverse Primer | AAAAAATCACTAATTTAACAATAAAA |
| Bisulfite PCR reaction #2 Forward Primer | TAATATTGAAATGATGAGTTAGG |
| Bisulfite PCR reaction #2 Reverse Primer | AAAAAACTCAAAATAAATTATACCT |
| Non-converted PCR Forward Primer | GTCGTGGTCAGTCCAACCAT |
| Non-converted PCR Reverse Primer | ATCTTCGAAGTGCGTCCTCC |

| **Reaction** | **Amplicon Size (bp)** |
| --- | --- |
| Bisulfite PCR reaction #1 | 1,986 |
| Bisulfite PCR reaction #2 | 1,706 |
| Non-converted PCR | 1,918 |

***SMRT sequencing***

In brief, pooled PCR amplicons were quantified using Qubit fluorometric analysis (Life Technologies) and a Bioanalysis 12000 chip (Agilent Technologies) to assess PCR amplicon quality, size, and quantity. Additionally, barcoded and pooled amplicons were purified using Ampure XP Solid Phase Reversible Immobilization (Beckman Coulter) at 0.8-fold volume. SMRTbell libraries were constructed using end-repair, ligation, and exonuclease purification strategies detailed in the Pacific Biosciences P5-C3 Template Preparation Kit protocols. SMRTbell templates were then bound to polymerase molecules for 4 hours at 25°C using 3 nM of the amplicon SMRTbell library and excess P5 DNA polymerase at a concentration of 9 nM as previously described.[2] The polymerase-template complexes were immobilized at 250 pM for 30 min on nanofabricated SMRT cells containing an array of zero-mode waveguides (ZMWs), and ZMWs were analyzed for sequencing to generate reads using a 1x180-minute collection protocol. Circular consensus sequencing (CCS) was employed using multiple passes on each SMRTbell to generate CCS reads with higher accuracy for data analysis. The “reads of insert” pipeline was utilized with a filter of 85% accuracy and 1-pass prior to downstream analyses.

**References**

1. Yong RL, Yang C, Lu J, Wang H, Schlaff CD, Tandle A, Graves CA, Elkahloun AG, Chen X, Zhuang Z, Lonser RR (2014) Cell transcriptional state alters genomic patterns of DNA double-strand break repair in human astrocytes. Nat Commun 5: 5799 doi:10.1038/ncomms6799

2. Rasko DA, Webster DR, Sahl JW, Bashir A, Boisen N, Scheutz F, Paxinos EE, Sebra R, Chin C-S, Iliopoulos D, Klammer A, Peluso P, Lee L, Kislyuk AO, Bullard J, Kasarskis A, Wang S, Eid J, Rank D, Redman JC, Steyert SR, Frimodt-Møller J, Struve C, Petersen AM, Krogfelt KA, Nataro JP, Schadt EE, Waldor MK (2011) Origins of the E. coli strain causing an outbreak of hemolytic-uremic syndrome in Germany. The New England journal of medicine 365: 709-717 doi:10.1056/NEJMoa1106920
